# Bis[4-methyl-2-(4-methyl­phenyldiazen­yl)phenolato-κ^2^ *N*,*O*]nickel(II)

**DOI:** 10.1107/S1600536809013920

**Published:** 2009-04-25

**Authors:** Dexin Guan, Hongjian Sun

**Affiliations:** a School of Chemistry and Chemical Engineering, Shandong University, Jinan 250100, People’s Republic of China

## Abstract

In the crystal structure of the title compound, [Ni(C_14_H_13_N_2_O)_2_], the Ni^II^ ion is located on an inversion center and is coordinated by two 4-methyl-2-(4-methyl­phenyl­diazen­yl)phenolate anions in a slightly distorted square-planar geometry. Within the anion, the two benzene rings are twisted from each other with a dihedral angle of 45.97 (12)°. No hydrogen bonding is found in the crystal structure.

## Related literature

For general background, see: Frey (2005 ▶).
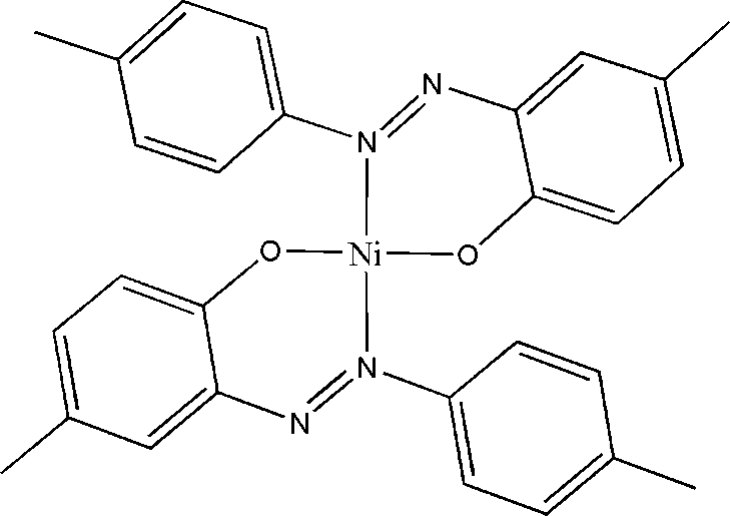


## Experimental

### 

#### Crystal data

[Ni(C_14_H_13_N_2_O)_2_]
*M* *_r_* = 509.24
Monoclinic, 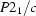

*a* = 9.5211 (10) Å
*b* = 10.8162 (11) Å
*c* = 12.2647 (13) Å
β = 105.367 (2)°
*V* = 1217.9 (2) Å^3^
*Z* = 2
Mo *K*α radiation
μ = 0.83 mm^−1^
*T* = 293 K
0.15 × 0.15 × 0.10 mm

#### Data collection

Bruker SMART APEX diffractometer
Absorption correction: multi-scan (*SADABS*; Sheldrick, 1996 ▶) *T*_min_ = 0.885, *T*_max_ = 0.920
7063 measured reflections
2775 independent reflections
1890 reflections with *I* > 2σ(*I*)
*R*_int_ = 0.032

#### Refinement

*R*[*F* ^2^ > 2σ(*F* ^2^)] = 0.038
*wR*(*F* ^2^) = 0.099
*S* = 1.02
2775 reflections
212 parameters
All H-atom parameters refined
Δρ_max_ = 0.24 e Å^−3^
Δρ_min_ = −0.19 e Å^−3^

### 

Data collection: *APEX2* (Bruker, 2008 ▶); cell refinement: *SAINT* (Bruker, 2008 ▶); data reduction: *SAINT*; program(s) used to solve structure: *SHELXS97* (Sheldrick, 2008 ▶); program(s) used to refine structure: *SHELXL97* (Sheldrick, 2008 ▶); molecular graphics: *ORTEP-3 for Windows* (Farrugia, 1997 ▶); software used to prepare material for publication: *WinGX* (Farrugia, 1999 ▶).

## Supplementary Material

Crystal structure: contains datablocks global, I. DOI: 10.1107/S1600536809013920/xu2508sup1.cif

Structure factors: contains datablocks I. DOI: 10.1107/S1600536809013920/xu2508Isup2.hkl

Additional supplementary materials:  crystallographic information; 3D view; checkCIF report

## Figures and Tables

**Table 1:** Selected bond lengths (Å)

Ni—O	1.8118 (16)
Ni—N1	1.8988 (18)

**Fig. 1.**
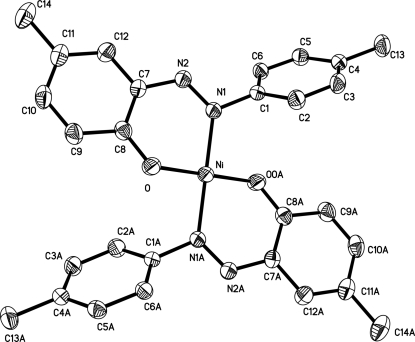
View of the title compound, showing 25% displacement ellipsoids. H atoms were omitted. Symmetry code: (A) 2 - x, 2 - y, -z.

## supplementary crystallographic information

### Comment

Nickel hydride complexes are one of the most valuable catalysts and intermediates. Frey (2005) has successfully synthesized a new type of nickel hydride Ni(H)(*ortho*-S—C_6_H_4_PPh_2_)(PMe_3_)_2_. In the previous work in our lab, we have reported similar reactions between nickel or cobalt hydrides and phenol derivates. So the reaction between Ni(H)(*ortho*-S—C_6_H_4_PPh_2_)(PMe_3_)_2_ and phenol derivates was carried out to explore the acidity of the hydrogen ligand. The title compound, as an unexpected compound, was synthesized.

The molecular structure is shown in Fig. 1. The Ni^II^ ion is located in an inversion center and coordinated by two 2-(4'-methylphenylazo)-4-methylphenol anions in a square-planar geometry (Table 1). No hydrogen bonding is found in the crystal structure.

### Experimental

Ni(H)(*ortho*-S-C_6_H_4_PPh_2_)(PMe_3_)_2_ (1.19 g, 2.35 mmol) and 2-(4'-methylphenylazo)-4-methylphenol (0.54 g, 2.38 mmol) was mixed in -80 °C. The mixture was stirred between -20 °C to 0 °C for 18 h and a red solution was formed. Green residue was filtered off, Then the solvent was removed in vacuum. The residue was extracted with pentane, and then diethyl ether. The extractions were kept in -20°C. The title compound was obtained from the pentane extractions as green crystals for X-ray diffraction.

### Refinement

The H atoms were geometrically placed and refined isotropically.

### Crystal data

**Table:**

[Ni(C_14_H_13_N_2_O)_2_]	*F*(000) = 532
*M**_r_* = 509.24	*D*_x_ = 1.389 Mg m^−^^3^
Monoclinic, *P*2_1_/*c*	Mo *K*α radiation, λ = 0.71073 Å
Hall symbol: -P 2ybc	Cell parameters from 1666 reflections
*a* = 9.5211 (10) Å	θ = 2.6–23.4°
*b* = 10.8162 (11) Å	µ = 0.83 mm^−^^1^
*c* = 12.2647 (13) Å	*T* = 293 K
β = 105.367 (2)°	Block, green
*V* = 1217.9 (2) Å^3^	0.15 × 0.15 × 0.10 mm
*Z* = 2	

### Data collection

**Table:**

Bruker SMART APEX diffractometer	2775 independent reflections
Radiation source: fine-focus sealed tube	1890 reflections with *I* > 2σ(*I*)
graphite	*R*_int_ = 0.032
ω scans	θ_max_ = 27.6°, θ_min_ = 2.2°
Absorption correction: multi-scan (*SADABS*; Sheldrick, 1996)	*h* = −12→11
*T*_min_ = 0.885, *T*_max_ = 0.920	*k* = −13→13
7063 measured reflections	*l* = −9→15

### Refinement

**Table:**

Refinement on *F*^2^	Primary atom site location: structure-invariant direct methods
Least-squares matrix: full	Secondary atom site location: difference Fourier map
*R*[*F*^2^ > 2σ(*F*^2^)] = 0.038	Hydrogen site location: inferred from neighbouring sites
*wR*(*F*^2^) = 0.099	All H-atom parameters refined
*S* = 1.02	*w* = 1/[σ^2^(*F*_o_^2^) + (0.0451*P*)^2^ + 0.1292*P*] where *P* = (*F*_o_^2^ + 2*F*_c_^2^)/3
2775 reflections	(Δ/σ)_max_ < 0.001
212 parameters	Δρ_max_ = 0.24 e Å^−^^3^
0 restraints	Δρ_min_ = −0.19 e Å^−^^3^

### Special details

**Table:**

Geometry. All e.s.d.'s (except the e.s.d. in the dihedral angle between two l.s. planes) are estimated using the full covariance matrix. The cell e.s.d.'s are taken into account individually in the estimation of e.s.d.'s in distances, angles and torsion angles; correlations between e.s.d.'s in cell parameters are only used when they are defined by crystal symmetry. An approximate (isotropic) treatment of cell e.s.d.'s is used for estimating e.s.d.'s involving l.s. planes.
Refinement. Refinement of *F*^2^ against ALL reflections. The weighted *R*-factor *wR* and goodness of fit *S* are based on *F*^2^, conventional *R*-factors *R* are based on *F*, with *F* set to zero for negative *F*^2^. The threshold expression of *F*^2^ > σ(*F*^2^) is used only for calculating *R*-factors(gt) *etc*. and is not relevant to the choice of reflections for refinement. *R*-factors based on *F*^2^ are statistically about twice as large as those based on *F*, and *R*- factors based on ALL data will be even larger.

### Fractional atomic coordinates and isotropic or equivalent isotropic displacement parameters (Å^2^)

**Table:**

	*x*	*y*	*z*	*U*_iso_*/*U*_eq_	
Ni	1.0000	1.0000	0.0000	0.04172 (15)	
N1	0.96662 (19)	0.83618 (16)	0.04286 (15)	0.0412 (4)	
N2	1.02859 (19)	0.77982 (17)	0.13462 (15)	0.0448 (5)	
O	1.0688 (2)	1.04670 (16)	0.14634 (14)	0.0552 (5)	
C7	1.1204 (2)	0.8429 (2)	0.22272 (18)	0.0428 (5)	
C1	0.8685 (2)	0.75494 (19)	−0.03615 (18)	0.0402 (5)	
C6	0.9169 (3)	0.6442 (2)	−0.0673 (2)	0.0440 (5)	
C4	0.6804 (3)	0.6040 (2)	−0.1919 (2)	0.0474 (6)	
C5	0.8231 (3)	0.5700 (2)	−0.1455 (2)	0.0480 (6)	
C2	0.7255 (3)	0.7908 (2)	−0.0813 (2)	0.0527 (6)	
C12	1.1914 (3)	0.7700 (3)	0.3162 (2)	0.0524 (6)	
C8	1.1345 (3)	0.9729 (2)	0.2279 (2)	0.0462 (6)	
C3	0.6333 (3)	0.7146 (2)	−0.1575 (2)	0.0559 (7)	
C9	1.2225 (3)	1.0238 (3)	0.3292 (2)	0.0571 (7)	
C11	1.2798 (3)	0.8206 (3)	0.4119 (2)	0.0560 (7)	
C10	1.2937 (3)	0.9492 (3)	0.4156 (2)	0.0595 (7)	
C13	0.5765 (5)	0.5215 (4)	−0.2756 (4)	0.0741 (10)	
C14	1.3546 (5)	0.7421 (5)	0.5127 (3)	0.0827 (11)	
H10	1.347 (3)	0.987 (2)	0.479 (2)	0.050 (7)*	
H5	0.862 (3)	0.494 (2)	−0.165 (2)	0.048 (7)*	
H6	1.014 (2)	0.624 (2)	−0.0350 (18)	0.047 (6)*	
H12	1.173 (3)	0.686 (2)	0.311 (2)	0.061 (8)*	
H3	0.541 (3)	0.737 (2)	−0.185 (2)	0.066 (8)*	
H9	1.232 (3)	1.111 (3)	0.333 (2)	0.066 (8)*	
H13B	0.594 (4)	0.440 (4)	−0.253 (3)	0.127 (16)*	
H13A	0.603 (5)	0.522 (4)	−0.338 (4)	0.127 (19)*	
H2	0.689 (3)	0.867 (3)	−0.057 (2)	0.076 (8)*	
H13C	0.491 (5)	0.541 (3)	−0.290 (3)	0.106 (15)*	
H14C	1.454 (4)	0.752 (4)	0.527 (3)	0.111 (13)*	
H14A	1.318 (4)	0.761 (4)	0.576 (3)	0.119 (14)*	
H14B	1.341 (5)	0.663 (5)	0.504 (4)	0.16 (2)*	

### Atomic displacement parameters (Å^2^)

**Table:**

	*U*^11^	*U*^22^	*U*^33^	*U*^12^	*U*^13^	*U*^23^
Ni	0.0496 (3)	0.0325 (2)	0.0400 (2)	−0.00195 (18)	0.00673 (18)	−0.00217 (18)
N1	0.0429 (10)	0.0346 (10)	0.0423 (10)	−0.0008 (8)	0.0046 (8)	−0.0015 (8)
N2	0.0455 (11)	0.0419 (11)	0.0439 (11)	0.0006 (8)	0.0062 (9)	0.0001 (9)
O	0.0800 (13)	0.0372 (8)	0.0422 (9)	−0.0034 (8)	0.0052 (9)	−0.0025 (8)
C7	0.0422 (12)	0.0466 (13)	0.0379 (12)	0.0001 (10)	0.0076 (10)	−0.0020 (10)
C1	0.0418 (13)	0.0339 (11)	0.0414 (12)	−0.0036 (9)	0.0047 (10)	0.0028 (9)
C6	0.0388 (13)	0.0380 (12)	0.0513 (14)	0.0008 (10)	0.0050 (10)	0.0000 (11)
C4	0.0496 (14)	0.0398 (13)	0.0466 (13)	−0.0069 (10)	0.0020 (11)	0.0021 (11)
C5	0.0507 (14)	0.0354 (13)	0.0547 (15)	0.0018 (11)	0.0085 (12)	−0.0051 (11)
C2	0.0449 (14)	0.0416 (14)	0.0648 (16)	0.0060 (11)	0.0029 (12)	−0.0021 (12)
C12	0.0568 (16)	0.0512 (16)	0.0472 (14)	0.0059 (13)	0.0103 (12)	0.0003 (12)
C8	0.0476 (14)	0.0508 (15)	0.0414 (13)	−0.0060 (10)	0.0142 (11)	−0.0070 (10)
C3	0.0388 (14)	0.0512 (15)	0.0672 (17)	0.0062 (12)	−0.0045 (12)	0.0028 (13)
C9	0.0657 (17)	0.0606 (18)	0.0435 (14)	−0.0125 (13)	0.0119 (13)	−0.0114 (12)
C11	0.0481 (14)	0.0743 (19)	0.0431 (14)	0.0056 (13)	0.0077 (11)	−0.0004 (13)
C10	0.0530 (16)	0.083 (2)	0.0392 (14)	−0.0108 (15)	0.0070 (12)	−0.0122 (14)
C13	0.067 (2)	0.061 (2)	0.075 (2)	−0.0100 (17)	−0.0148 (19)	−0.0089 (17)
C14	0.074 (3)	0.112 (4)	0.0518 (19)	0.013 (2)	−0.0005 (17)	0.013 (2)

### Geometric parameters (Å, °)

**Table:**

Ni—O^i^	1.8118 (16)	C2—C3	1.374 (3)
Ni—O	1.8118 (16)	C2—H2	0.98 (3)
Ni—N1	1.8988 (18)	C12—C11	1.364 (3)
Ni—N1^i^	1.8988 (18)	C12—H12	0.92 (2)
N1—N2	1.278 (2)	C8—C9	1.413 (3)
N1—C1	1.451 (3)	C3—H3	0.89 (2)
N2—C7	1.377 (3)	C9—C10	1.361 (4)
O—C8	1.303 (3)	C9—H9	0.95 (3)
C7—C12	1.408 (3)	C11—C10	1.397 (4)
C7—C8	1.412 (3)	C11—C14	1.513 (4)
C1—C6	1.373 (3)	C10—H10	0.91 (3)
C1—C2	1.382 (3)	C13—H13B	0.92 (4)
C6—C5	1.381 (3)	C13—H13A	0.87 (5)
C6—H6	0.93 (2)	C13—H13C	0.81 (4)
C4—C5	1.377 (3)	C14—H14C	0.92 (4)
C4—C3	1.382 (3)	C14—H14A	0.95 (4)
C4—C13	1.514 (4)	C14—H14B	0.86 (5)
C5—H5	0.95 (2)		
			
O^i^—Ni—O	180.000 (1)	C11—C12—H12	121.2 (16)
O^i^—Ni—N1	88.33 (8)	C7—C12—H12	116.8 (16)
O—Ni—N1	91.67 (8)	O—C8—C7	123.7 (2)
O^i^—Ni—N1^i^	91.67 (8)	O—C8—C9	119.2 (2)
O—Ni—N1^i^	88.33 (8)	C7—C8—C9	117.0 (2)
N1—Ni—N1^i^	180.0	C2—C3—C4	121.8 (2)
N2—N1—C1	111.38 (17)	C2—C3—H3	119.1 (17)
N2—N1—Ni	128.07 (14)	C4—C3—H3	119.2 (17)
C1—N1—Ni	120.38 (13)	C10—C9—C8	120.7 (3)
N1—N2—C7	120.24 (19)	C10—C9—H9	122.2 (16)
C8—O—Ni	124.29 (15)	C8—C9—H9	117.0 (16)
N2—C7—C12	115.5 (2)	C12—C11—C10	117.3 (2)
N2—C7—C8	124.1 (2)	C12—C11—C14	121.8 (3)
C12—C7—C8	120.1 (2)	C10—C11—C14	120.8 (3)
C6—C1—C2	120.0 (2)	C9—C10—C11	122.8 (3)
C6—C1—N1	120.67 (19)	C9—C10—H10	117.0 (15)
C2—C1—N1	119.3 (2)	C11—C10—H10	120.2 (14)
C1—C6—C5	119.8 (2)	C4—C13—H13B	109 (2)
C1—C6—H6	117.3 (15)	C4—C13—H13A	108 (3)
C5—C6—H6	122.9 (15)	H13B—C13—H13A	101 (3)
C5—C4—C3	118.0 (2)	C4—C13—H13C	115 (3)
C5—C4—C13	121.3 (3)	H13B—C13—H13C	114 (3)
C3—C4—C13	120.8 (3)	H13A—C13—H13C	108 (4)
C4—C5—C6	121.2 (2)	C11—C14—H14C	109 (2)
C4—C5—H5	121.9 (15)	C11—C14—H14A	111 (2)
C6—C5—H5	116.9 (15)	H14C—C14—H14A	113 (3)
C3—C2—C1	119.2 (2)	C11—C14—H14B	115 (3)
C3—C2—H2	120.0 (15)	H14C—C14—H14B	104 (4)
C1—C2—H2	120.8 (15)	H14A—C14—H14B	104 (4)
C11—C12—C7	122.0 (3)		

Symmetry codes: (i) −*x*+2, −*y*+2, −*z*.

## References

[bb1] Bruker (2008). *APEX2* and *SAINT* Bruker AXS Inc., Madison, Wisconsin, USA.

[bb2] Farrugia, L. J. (1997). *J. Appl. Cryst.* **30**, 565.

[bb3] Farrugia, L. J. (1999). *J. Appl. Cryst.* **32**, 837–838.

[bb4] Frey, M. (2005). PhD thesis, Darmstadt University of Technology, Germany.

[bb5] Sheldrick, G. M. (1996). *SADABS* University of Göttingen, Germany.

[bb6] Sheldrick, G. M. (2008). *Acta Cryst.* A**64**, 112–122. 10.1107/S0108767307043930 18156677

